# Flourine-18 Fluorodeoxyglucose Positron Emission Tomography/Computed Tomography Imaging of Peritoneal Carcinomatosis in Lung Cancer: A Case Study

**DOI:** 10.1055/s-0044-1778708

**Published:** 2024-06-27

**Authors:** Tshimogo Sathekge, Raef R. Boktor, Salvatore Berlangieri, Sze Ting Lee

**Affiliations:** 1 Faculty of Health Science, University of Witwatersrand, Johannesburg, South Africa; 2 Department of Molecular Imaging and Therapy, Austin Health, Melbourne, VIC, Australia; 3 Olivia Newton-John Cancer Research Institute, Melbourne, VIC, Australia; 4 School of Cancer Medicine, La Trobe University, Melbourne, VIC, Australia; 5 Faculty of Medicine, University of Melbourne, Melbourne, VIC, Australia

**Keywords:** ^18^ F-FDG PET/CT, peritoneal carcinomatosis, lung cancer, NSCLC, peritoneal biopsy

## Abstract

Lung cancer with peritoneal carcinomatosis (PC) is a rare disease presentation. The presence of peritoneal disease is a sign of poor prognosis and is hard to diagnose. Flourine-18 fluorodeoxyglucose positron emission tomography/computed tomography ( ^18^ F-FDG PET/CT) is becoming more clinically significant in the management of patients with PC. A 60-year-old male presented with nonsmall cell lung cancer (NSCLC) and later showed signs of peritoneal disease on ^18^ F-FDG PET/CT imaging, which subsequently lead to the diagnoses of PC with histopathology from peritoneal biopsy. The patient showed an excellent initial response to their NSCLC treatment but later presented with PC that was shown by FDG-avid ascites and a soft tissue mass in the pelvic area. The abdominal-pelvic lesions were confirmed cytologically to be peritoneal metastatic disease. ^18^ F-FDG PET/CT demonstrated value in preoperatively directing biopsy for diagnosing PC in this case of NSCLC. Further, ^18^ F-FDG PET/CT was useful in the monitoring of disease progression and thus influenced management in this case of NSCLC with PC, which is often challenging to detect and manage.

## Introduction

Lung cancer is the second most prevalent malignancy in the world, where a significant number of patients present with metastasis at the time of diagnosis contributing to high mortality. 1 The most common sites of metastasis in lung cancer are the skeletal system, adrenal glands, liver, brain, pleura, and distant lymph nodes. Progression of the primary cancer to the peritoneum is defined as peritoneal carcinomatosis (PC). In the setting of lung cancer, PC is a rare clinical event with a prevalence of 1.2%. 2 Despite PC in nonsmall cell lung cancer (NSCLC) being a rare clinical event, metastasis of lung cancer to the peritoneum at autopsy is not as rare with a prevalence of 2.7 to 16%. 3 This describes the need to utilize imaging and diagnostic tools in identifying PC.

Patients with PC have a significantly worse quality of life. 4 This includes symptoms of abdominal pain, bloating, shortness of breath as well as nausea. NSCLC patients with PC have a poor prognosis with a median overall survival of 5.2 months. However, the median overall survival in NSCLC with different underlying genetic subtypes varies due to the different treatment modalities available for different genetic mutations. This can be attributed to the treatment modalities available for different genetic mutations, whereby epidermal growth factor receptor (EGFR) mutation treatment regimens have shown more promising results compared with EGFR wild-type mutations. Reactive oxygen species-(ROS-1) positive (EGFR wild-type) patients, however, do seem to benefit from crizotinib that is considered an advance in NSCLC treatment. 4

Conventional imaging modalities like computer tomography (CT) have a limited sensitivity in detecting PC. The use of ^18^ F-FDG PET showed greater sensitivity as well as higher positive predicative value compared with CT. 5 It is shown that ^18^ F-FDG PET provides value to conventional imaging in detecting peritoneal disease and thus more effectively guiding peritoneal biopsy in the diagnosis of PC.

## Clinical Presentation

**Fig. 1:**
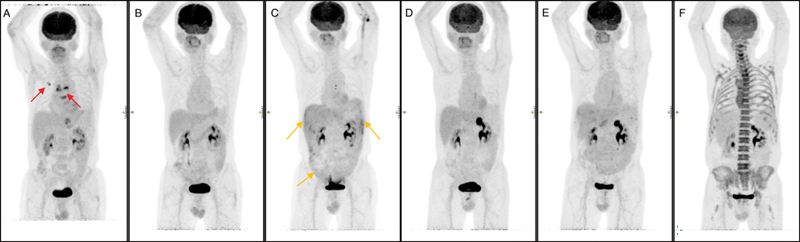
Series of maximum intensity projection images showing ( **A** ) disease at initial presentation ( *red arrows* ). ( **B** ) Post first line of treatment. ( **C** ) First peritoneal recurrence in the right and left upper abdominal quadrant and the vesicorectal pouch ( *orange arrows* ). ( **D** ) Complete metabolic response of the peritoneal diseases after second line treatment. ( **E** ) Further peritoneal recurrence in the mid abdomen. ( **F** ) complete metabolic response of the peritoneal disease after third line of treatment.

**Fig. 2:**
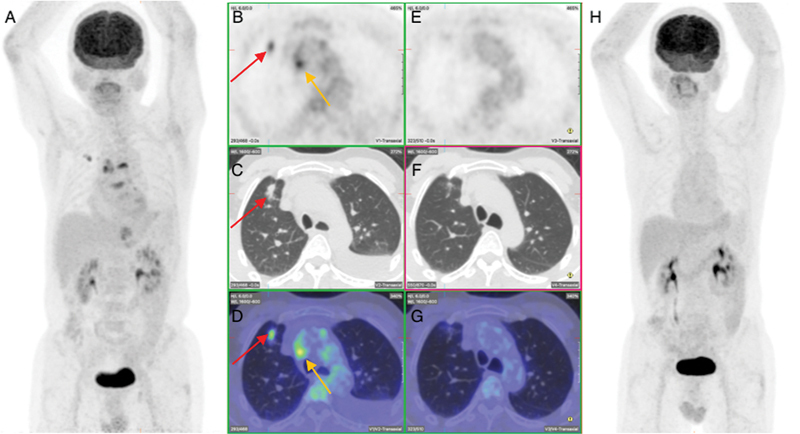
Pre- and post-treatment fluorodeoxyglucose positron emission tomography (PET) at first presentation showing complete metabolic response of primary right lung, nodal metastasis and left pleural effusion. ( **A** ). Pre-therapy maximum intensity projection (MIP) image showing right lung primary tumor and mediastinal nodal metastasis. ( **B** – **D** ) Pre-therapy transverse section of PET, computed tomography (CT), and fused images, respectively, showing the lung ( *red arrows* ) and nodal metastasis ( *orange arrows* ). ( **E** – **G** ) Post-therapy transverse section of PET, CT, and fused images, respectively, showing complete resolution of primary lung and nodal disease. ( **H** ) Post-therapy MIP image showing complete metabolic response. There was no evidence of FDG-avid local lung carcinoma recurrence or extra peritoneal spread. This led to a change to a 3rd line regimen which consisted of Carboplatin (AUC5), pemetrexed, atezolizumab, bevacizumab. Three months later 18F-FDG PET/CT showed excellent response with complete metabolic remission and no evidence of residual or new FDG-avid malignant or metastatic disease.

A 60-year-old male nonsmoker was diagnosed with stage IV ROS-1 positive NSCLC in the upper right lung. At the time of the first presentation, the patient presented with a pericardial tamponade requiring intensive care unit admission, which was subsequently drained and found to have positive cytology for NSCLC. Initial baseline PET/CT showed intense FDG avid mediastinal lymph nodes Fig 1A , Figs 2A–D .

Following this diagnosis, the patient commenced treatment with crizotinib, where the patient showed a positive response, achieving complete metabolic remission Fig. 1B , Fig 2E–G . A year later the patient underwent a ^18^ F-FDG PET/CT scan for the evaluation of disease reoccurrence due to the development of abdominal symptoms. The ^18^ F-FDG PET/CT showed ascites in the abdomen and pelvis with heterogenous low-grade FDG uptake and fat stranding, raising the possibility of peritoneal metastatic disease. Furthermore, there was a new large, intensely FDG-avid irregularly shaped soft tissue mass between the rectum and urinary bladder in keeping with a large peritoneal deposit. The patient underwent a biopsy that confirmed PC. Following this diagnosis, the treatment was changed to Lorlatinib.

Post-treatment ^18^ F-FDG PET/CT showed complete metabolic resolution of the ascites and peritoneal disease. No FDG-avid local lung recurrence or other metastasis indicated complete metabolic remission Fig 1D .

Nine months later the patient developed recurrent significant abdominal bloating and pain for which he underwent a ^18^ F-FDG PET/CT scan. Scan findings were consistent with recurrent peritoneal disease. There was no evidence of FDG-avid local lung carcinoma recurrence or extra peritoneal spread. This led to a change to a third line regimen that consisted of carboplatin (AUC5), pemetrexed, atezolizumab, and bevacizumab. Three months later ^18^ F-FDG PET/CT showed excellent response with complete metabolic remission and no evidence of residual or new FDG-avid malignant or metastatic disease Fig. 1F , Fig. 4E–H .

## Discussion

NSCLC is the most common lung cancer to present with peritoneal metastasis. 4 The difference in detection rates of peritoneal metastasis clinically compared with autopsy can in part be attributed to the asymptomatic early presentation of peritoneal metastasis, as well as the difficulty to accurately diagnose PC. 4 Some of these challenges emphasize the value of using technologies such as ^18^ F-FDG PET/CT that effectively demonstrate image finding suggestive of peritoneal disease and can thus allow for more effective and accurate use of peritoneal biopsies to confirm the diagnosis PC. 6 Our case presentation demonstrates a unique perspective of using ^18^ F-FGD PET/CT to detect image findings suggestive of peritoneal disease that would ultimately lead to the effective use of peritoneal biopsy to histologically confirm the diagnosis of PC in a timeous manner. The image findings suggestive of peritoneal disease using ^18^ F-FDG PET/CT allowed for the early histological confirmation of diagnosis of PC, through peritoneal biopsy. This early confirmation of PC positively influenced the prognosis of our patient, due to the early commencement of treatment.

There is a growing use of ^18^ F-FDG PET/CT scans in metastatic peritoneal carcinomas. ^18^ F-FDG PET/CT has shown superior sensitivity compared with conventional CT scans. Standalone ^18^ F-FDG PET showed sensitivity of 57% as compared with 43% for standalone CT. Most notably, using ^18^ F-FDG PET in addition to CT scans increased sensitivity by a further 78.0% more than any of the individual modalities could achieve alone. When ^18^ F-FDG PET is used in addition to CT scans, a 95% positive predictive value for PC is demonstrated. 5

While there is evidence showing the improved utility of ^18^ F-FDG PET/CT in PC across numerous different types of primary cancers as seen by a meta-analysis which comprised of 14 studies and 671 patients that were analyzed. 7 However, notably none of these studies include a case of primary lung cancer. This shows the uniqueness of our patients' case, as the use of ^18^ F-FDG PET/CT to detect PC in lung cancer is not well described in literature.

Laparotomy with peritoneal biopsy is considered the gold standard for the diagnosis of PC due to its ability to visualize and physically assess the peritoneum. 8 Laparoscopic techniques are often used for staging patients; however, due to the invasiveness of this modality there is limited utility in monitoring disease progression with patient follow-ups. 8 While CT imaging is the current modality of choice preoperatively, it provides limited sensitivity due its dependence on variables such as tumor morphology, site, and size. 5 This is particularly important in the setting of PC as peritoneal tumor deposits tend to be of a smaller size and may be less numerous with sheet spread pattern as well as have variable presentations morphologically. 5 This variability in presentation, site, and size is thought to play a significant role in the diagnostic difficulty of PC. As shown in our case, ^18^ F-FDG PET/CT can be of value in the preoperative imaging of peritoneal disease to assist in the diagnosis of PC and avoid the invasive laparotomy in similar cases.

Timeous diagnosis of PC is of significance as less numerous deposits of peritoneal disease could make the patient more suited for cytoreduction preceding surgery. Disease progression and monitoring of response to chemotherapy are vital in the management of patients. This case demonstrates the ability of ^18^ F-FDG PET/CT to provide image finding that is suggestive of responses to chemotherapy, and thus aids in assessing disease progression as well as the management of a patient with PC.

^18^ F-FDG PET/CT aids in directing treatment plans and change of treatment modalities—in particular it can guide the use of tyrosine kinase inhibitors (TKI) in patients with NSCLC that can help improve patient survival. Tani et al found that NSCLC patients with PC receiving TKI treatment achieve a median overall survival of 20.3 months compared with 3.5 months when patients only received palliative care. 4 This has also been demonstrated in our case, as the use of ^18^ F-FDG PET/CT directed clinicians to use TKI that allowed our patient to achieve a complete metabolic response and hence helped improve patient's survival.

There are numerous limitations and drawbacks when ^18^ F-FDG PET/CT is used to detect PC in NSCLC. The limited spatial resolution of ^18^ F-FDG PET/CT may impede the detection of peritoneal metastasis as most peritoneal tumor implants are small nodules. Additionally, the peritoneal cavity contains organs like the liver and gastrointestinal tract that all have high physiological FDG uptake, thus making it difficult to distinguish normal uptake from that of PC. In view of these limitations, it is important to note the exciting new studies that demonstrate the utility of fluorodeoxyglucose positron emission tomography/magnetic resonance imaging (FDG PET/MRI), whereby it is found that FDG PET/MRI shows superior sensitivity and accuracy to FDG PET/CT in detecting PC. 9 FDG PET/MRI has the potential to provide more comprehensive and accurate information for the evaluation of PC in NSCLC patients, thus improving clinical decision-making and patient management.

This case demonstrates the value of ^18^ F-FDG PET/CT in the evaluation and management of NSCLC patients with PC. ^18^ F-FDG PET/CT detection of peritoneal disease notified timeous change of initial treatment that significantly improved our patients symptoms, metabolic response, and prolonged patient's survival.

## Conclusion

^18^ F-FDG PET/CT demonstrated value in preoperatively directing biopsy for diagnosing PC in this case of NSCLC. Further, ^18^ F-FDG PET/CT was useful in the monitoring of disease progression, thus influencing management in this case of NSCLC with PC, which is often challenging to detect and manage.

**Fig. 3:**
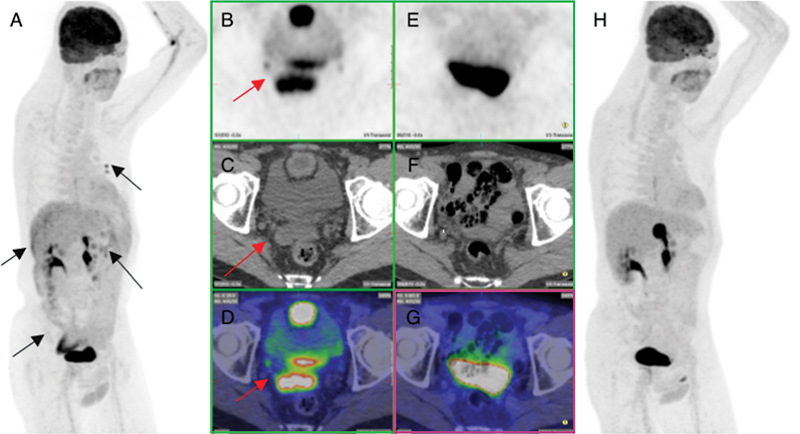
Pre- and post-treatment fluorodeoxyglucose positron emission tomography (PET) after first peritoneal recurrence showing complete metabolic response of peritoneal recurrence in the right and left upper abdomen and vesicorectal pouch. ( **A** ) Pre-therapy maximum intensity projection (MIP) image showing recurrence in the rectovesical pouch ( *black arrow* ), right and left upper abdominal quadrant, and mediational nodes ( *black arrows* ). ( **B** – **D** ) Pre-therapy transverse section of PET, computed tomography (CT) and fused images, respectively, showing mass in the rectovesical pouch ( *red arrow* ). ( **E** – **G** ) Post-therapy transverse section of PET, CT, and fused images, respectively, showing complete resolution of rectovesical mass. ( **H** ) Post-therapy MIP image showing complete metabolic response of the peritoneal recurrence and rectovesical mass.

**Fig. 4:**
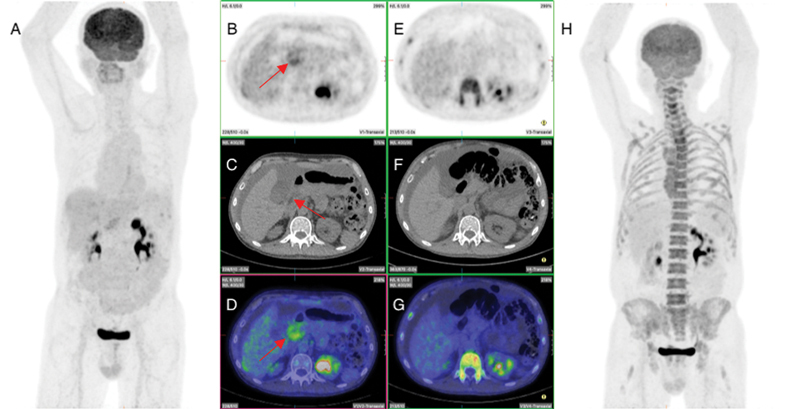
Pre- and post-treatment fluorodeoxyglucose positron emission tomography (PET) after second peritoneal recurrence showing complete metabolic response of peritoneal recurrence in the mid abdomen. ( **A** ) Pre-therapy maximum intensity projection (MIP) image showing recurrence in the mid abdomen. ( **B** – **D** ) Pre-therapy transverse section of PET, computed tomography (CT), and fused images, respectively, showing peritoneal recurrence in the mid abdomen and ascites ( *red arrow* ). ( **E** – **G** ) Post-therapy transverse section of PET, CT, and fused images, respectively, showing complete metabolic resolution of mid abdomen peritoneal disease but persistent ascites. ( **H** ) Post-therapy MIP image showing complete metabolic response of the peritoneal recurrence.
